# Comprehensive Pathogen Spectrum Analysis Using mNGS in AIDS Patients With Pulmonary Infections: Diagnostic Value and Clinical Implications

**DOI:** 10.1093/ofid/ofag395

**Published:** 2026-07-13

**Authors:** Wu Liang, Liu Tingting, Liu Ying, Wang Sa, Zhang Yuanyuan, Zhao Hongxin

**Affiliations:** National Center for Infectious Diseases, Beijing Ditan Hospital, Capital Medical University, Beijing, China; Clinical Center for HIV/AIDS, Beijing Ditan Hospital, Capital Medical University, Beijing, China; National Key Laboratory of Intelligent Tracking and Forecasting for Infectious Diseases, Beijing Ditan Hospital, Capital Medical University, Beijing, China; Beijing Institute of Infectious Diseases, Beijing Ditan Hospital, Capital Medical University, Beijing, China; National Center for Infectious Diseases, Beijing Ditan Hospital, Capital Medical University, Beijing, China; Clinical Center for HIV/AIDS, Beijing Ditan Hospital, Capital Medical University, Beijing, China; National Center for Infectious Diseases, Beijing Ditan Hospital, Capital Medical University, Beijing, China; Clinical Center for HIV/AIDS, Beijing Ditan Hospital, Capital Medical University, Beijing, China; National Key Laboratory of Intelligent Tracking and Forecasting for Infectious Diseases, Beijing Ditan Hospital, Capital Medical University, Beijing, China; Beijing Institute of Infectious Diseases, Beijing Ditan Hospital, Capital Medical University, Beijing, China; National Center for Infectious Diseases, Beijing Ditan Hospital, Capital Medical University, Beijing, China; Clinical Center for HIV/AIDS, Beijing Ditan Hospital, Capital Medical University, Beijing, China

**Keywords:** AIDS-related pulmonary infections, diagnostic accuracy, metagenomic next-generation sequencing (mNGS), mixed infections, opportunistic pathogens

## Abstract

**Background:**

This study evaluated the diagnostic value of metagenomic next-generation sequencing (mNGS) in identifying pathogens causing pulmonary infections in 64 acquired immunodeficiency syndrome (AIDS) patients at Beijing Ditan Hospital.

**Methods:**

Bronchoalveolar lavage fluid (BALF) samples were analyzed using mNGS and conventional microbiological tests (CMT). Diagnostic performance was compared, and random forest analysis was used to assess pathogenicity.

**Results:**

mNGS detected 45 pathogens, including 14 viruses, 3 fungi, and 28 bacteria. Compared with CMT, mNGS showed higher sensitivity for detecting bacteria (75.0% vs 29.17%), fungi (45.0% vs 16.67%), and viruses (80.0% vs 20.83%). Mixed infections were identified in 55.2% of cases, predominantly *Pneumocystis pneumonia* (PCP) with bacterial coinfections. However, mNGS had lower concordance with CMT for viruses (24.1% for cytomegalovirus) and *Mycobacterium tuberculosis* (75.0%). Random forest analysis highlighted *Candida albicans* and *Stenotrophomonas maltophilia* as highly pathogenic.

**Conclusions:**

While mNGS demonstrated superior broad-spectrum detection, its limitations in viral and TB diagnosis underscore the need for optimized protocols. The study supports mNGS as a complementary tool for diagnosing complex pulmonary infections in AIDS patients, enhancing precision medicine but requiring further refinement for widespread clinical adoption.

With the widespread use of antiretroviral therapy (ART) and continuous optimization of treatment regimens, the mortality rate of individuals infected with the human immunodeficiency virus (HIV-1) has shown a declining trend. However, among people living with HIV (PLWH), acquired immunodeficiency syndrome (AIDS)-related deaths remain the leading cause of infectious disease mortality, with opportunistic infections (OIs) being the primary contributor to patient deaths [1]. Among these, respiratory infections, particularly pulmonary infections, exhibit the highest incidence [2]. Timely and accurate identification of pathogens and targeted treatment are crucial for improving efficacy and prognosis. However, traditional pathogen detection methods often have several limitations, including prolonged turnaround time, limited sensitivity, and restricted pathogen coverage, which may constrain their utility in clinical precision medicine [3–5].

In recent years, metagenomic next-generation sequencing (mNGS) has emerged as a novel technological support for pathogen diagnosis [6]. While conventional microbiological testing (CMT) and pathological examinations can identify only a proportion of causative pathogens, mNGS offers broader pathogen spectrum coverage. However, the clinical relevance of some detected microorganisms remains uncertain, and the interpretation of mNGS results requires careful correlation with clinical findings and conventional microbiological testing [6]. Nevertheless, mNGS has limitations in identifying pathogens against the complex background of respiratory commensal microbiota, and its clinical application has not yet been fully validated in either immunocompromised or immunocompetent patients. Moreover, current evidence regarding the pathogen spectrum of pulmonary infections and the clinical interpretation of mNGS results in severely immunocompromised AIDS patients remains limited, particularly when bronchoalveolar lavage fluid (BALF) samples are used [7–9].

This study aims to utilize mNGS technology to analyze bronchoalveolar lavage fluid (BALF) samples from AIDS patients with pulmonary infections. By integrating clinical and CMT results, we seek to provide a more rational interpretation of mNGS reports, analyze the clinical disease spectrum of AIDS-related pulmonary infections, and compare the correlations and differences among mNGS disease spectra, CMT disease spectra, and clinically diagnosed disease spectra. The goal is to evaluate the clinical utility of mNGS in the diagnosis of opportunistic infections in AIDS patients and to compare its performance with conventional microbiological testing.

## METHODS

### Study Subjects

This study enrolled 64 hospitalized patients diagnosed with AIDS and pulmonary infections at Beijing Ditan Hospital, Capital Medical University, between August 2022 and August 2024. The diagnosis of pulmonary infections was confirmed through rounds of evaluations by senior physicians. Within 48–72 hours of admission, patients underwent bronchoscopy, and BALF samples were collected for both mNGS and conventional microbiological tests, including bacterial cultures, immunological assays, polymerase chain reaction (PCR), and pathological examinations. Clinical trial number: not applicable.

### Pathogen Identification and Disease Spectrum Analysis

**Figure 1.**
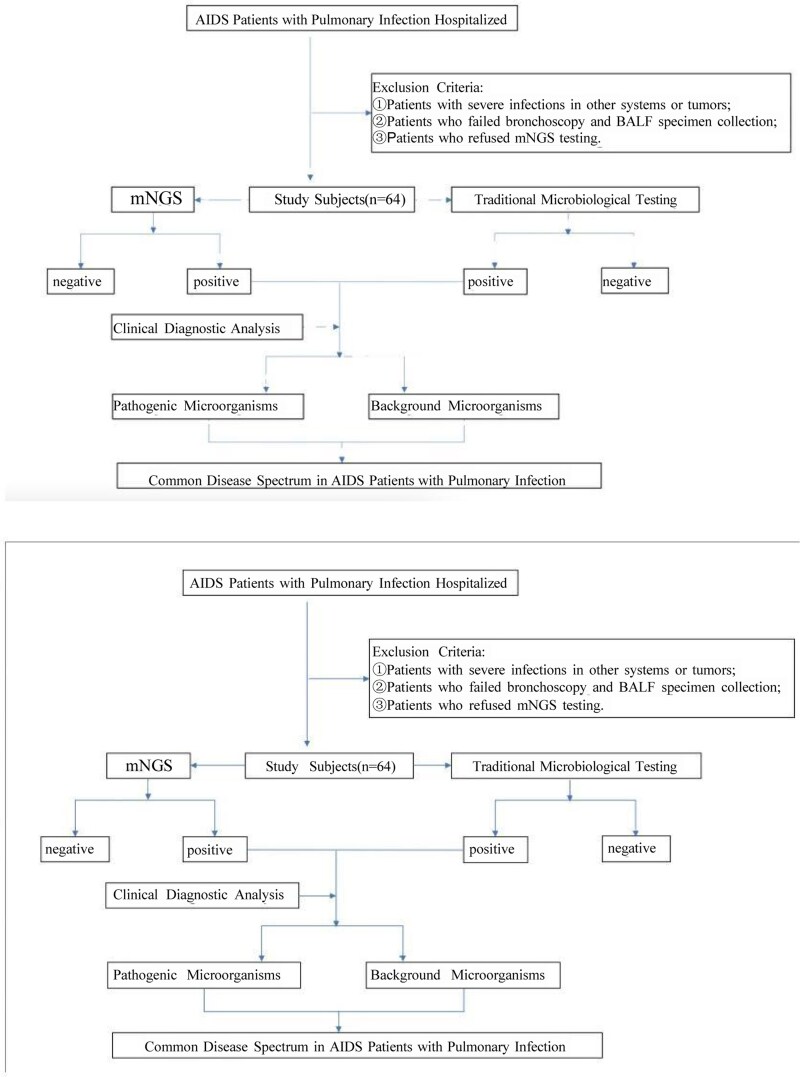
Technical workflow diagram of mNGS pathogen spectrum analysis in AIDS patients with pulmonary infections.

Pathogens detected by mNGS were classified as pathogenic if validated by clinical diagnosis or traditional microbiological methods; otherwise, they were considered commensal or colonizing background microorganisms. This study analyzed the correlations and differences among mNGS disease spectra (Figure 1), CMT disease spectra, and clinically diagnosed disease spectra in AIDS patients with pulmonary infections.

### Data Collection

Data were extracted from electronic medical records, including demographic information, disease duration, duration of antiretroviral therapy (ART), length of hospitalization, types of respiratory support, clinical outcomes, and diagnoses.

Laboratory data were systematically collected and included routine blood tests (eg, white blood cell count, neutrophil count, lymphocyte count, hemoglobin, and platelet count), biochemical markers (eg, liver and renal function tests, lactate dehydrogenase, and electrolytes), infection-related indicators (eg, C-reactive protein [CRP], procalcitonin [PCT], and erythrocyte sedimentation rate [ESR]), arterial blood gas parameters, and immunological indices (eg, CD4+ T-cell count and CD8+ T-cell count). In addition, results from polymerase chain reaction (PCR), bacterial and fungal cultures, pathological examinations, and chest computed tomography (CT) imaging were collected.

Metagenomic next-generation sequencing (mNGS) results were also retrieved, covering detected pathogens including bacteria, fungi, viruses, and parasites.

### Statistical Analysis

Data were analyzed using SPSS 22.0. Normally distributed continuous variables were expressed as mean ± standard deviation (*X* ± *S*), while nonnormally distributed data were described using medians and interquartile ranges. Categorical variables were presented as frequencies or rates, with group comparisons performed using chi-square tests. The correlation between CMT and mNGS disease spectra was assessed using kappa consistency analysis. In addition, random forest analysis was performed to evaluate the relative importance of variables associated with group classification, and Mantel tests were conducted to assess the correlation between clinical variables and microbial profile data. These analyses were included as exploratory approaches to further investigate variable contributions and clinicomicrobiological associations. A *P*-value <.05 was considered statistically significant.

## RESULTS

### Baseline Characteristics

The study included 64 AIDS patients with pulmonary infections, of whom 62 (96.88%) were male. The majority (56.25%) were aged 18–45, while 43.75% were aged 46–69. A smoking history was reported in 39.06% of patients. HIV duration was ≤3 months in 57.81% of cases, and 62.5% had either not received ART or had discontinued treatment. Pulmonary infection duration was ≤2 weeks in 29.69%, 2–8 weeks in 42.19%, and >8 weeks in 28.13%. Hospitalization length was ≤2 weeks in 35.94%, 2–4 weeks in 43.75%, and >4 weeks in 20.31%. The majority (87.50%) showed improvement, while 12.50% showed no clinical improvement at discharge or died during hospitalization (Table 1).

**Table 1.** Demographic and Clinical Characteristics of AIDS Patients With Pulmonary Infections

Category	Subgroup	n	%
Sex	Male	62	96.88%
	Female	2	3.13%
Age (years)	18–45	36	56.25%
	46–69	28	43.75%
Smoking	Yes	25	39.06%
	No	39	60.94%
HIV duration	≤3 m	37	57.81%
	>3 m	27	42.19%
ART status	None (discontinued)	40 (6)	62.50% (9.37%)
	≤3 m	10	15.63%
	>3 m	14	21.88%
Pulmonary infection duration	≤2 wks	19	29.69%
	2–8 wks	27	42.19%
	>8 wks	18	28.13%
Hospital stay	≤2 wks	23	35.94%
	2–4 wks	28	43.75%
	>4 wks	13	20.31%
Outcome	Improved	56	87.50%
	Unimproved/death	8	12.50%
Clinical diagnoses (n = 64)	PCP	35	54.69%
	Bacterial pneumonia	31	48.44%
	Fungal pneumonia	19	29.69%
	Tuberculosis (TB)	15	23.44%
	CMV pneumonia	12	18.75%
	Others	6	9.38%
Etiological diagnoses with pathogen identified by mNGS and/or conventional microbiological tests (CMT) (n = 48, 75.0%)	PCP	16	33.33%
	Bacterial	14	29.17%
	CMV	10	20.83%
	TB	10	20.83%
	NTM	8	16.67%
	Fungal	8	16.67%
	Others	4	8.33%
Respiratory support	None	21	32.81%
	Nasal cannula	25	39.06%
	Face mask	2	3.13%
	Reservoir mask	7	10.94%
	HFNC	5	7.81%
	Invasive ventilation	4	6.25%

Other clinical diagnoses included nontuberculous mycobacterial (NTM) infection, Kaposi's sarcoma, and a small number of cases with nonspecific interstitial pneumonia or drug-related lung injury in which no specific pathogen was identified. HFNC, high-flow nasal cannula.

Abbreviation: HFNC, high-flow nasal cannula.

The length of hospitalization was divided into 3 groups: <2 weeks, 2–4 weeks, and >4 weeks. Patients with CD4 ≥ 200 cells/μL, HIV RNA < 20 copies/mL, and serum Cl^−^ ≥ 99 mmol/L had a shorter hospital stay compared to those with CD4 < 200 cells/μL, HIV RNA ≥100 000 copies/mL, and serum Cl^−^ < 99 mmol/L.

Additionally, patients with arterial blood gas PaO_2_ ≥ 60 mmHg and neutrophil percentage (NE%) ≤ 75% had a better prognosis than those with PaO_2_ < 60 mmHg and NE% > 75%.

### Clinical and Pathological Diagnoses

The clinical disease spectrum included *Pneumocystis pneumonia* (PCP) (54.69%), bacterial pneumonia (48.44%), fungal pneumonia (29.69%), tuberculosis (TB) (23.44%), and cytomegalovirus (CMV) pneumonia (18.75%). The CMT pathogen spectrum identified PCP (33.33%), bacterial pneumonia (29.17%), CMV pneumonia (20.83%), tuberculosis (20.83%), nontuberculous mycobacteria (16.67%), and fungal pneumonia (16.67%).

### Mixed Infections and Diagnostic Consistency

Coinfections were clinically diagnosed in 55.2% of patients, with the most frequent combinations being PCP with bacterial infection (25.9%), PCP with CMV infection (19.0%), and PCP with fungal infection (19.0%). Other observed patterns included bacterial-fungal coinfections (15.5%), *Mycobacterium tuberculosis* (MTB) with bacterial infection (10.3%), and MTB-PCP coinfections (3.4%). To evaluate diagnostic accuracy, we performed comprehensive pathogen detection using droplet digital PCR (ddPCR), which simultaneously tested for 10 respiratory viruses, 11 bacterial species, 5 fungal species, and 2 atypical pathogens (*M. tuberculosis* and *Chlamydia psittaci*).

**Figure 2.**
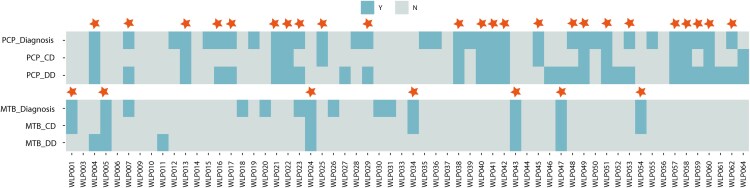
Consistency between clinical diagnosis of PCP/MTB and CMT/ddPCR testing. (Note: “Y” indicates a positive test result, “N” indicates a negative test result; the red star ★ indicates concordance between clinical diagnosis and comprehensive CMT testing).

Comparative analysis revealed significantly improved diagnostic concordance for PCP when combining clinical assessment with CMT/ddPCR testing (72.7%, 24/33 cases) versus CMT alone (45.5%), representing a 27.3% absolute improvement. Marked concordance (★) was observed in these aligned cases. In contrast, no diagnostic improvement was seen for MTB, with both CMT alone and CMT/ddPCR showing 50.0% concordance (7/14 cases) with clinical diagnosis. These findings suggest that ddPCR enhances detection accuracy for PCP but not for MTB, potentially reflecting limitations in current MTB diagnostic approaches (Figure 2).

### mNGS Pathogen Detection

#### Pathogen Detection Results by mNGS

**Figure 3.**
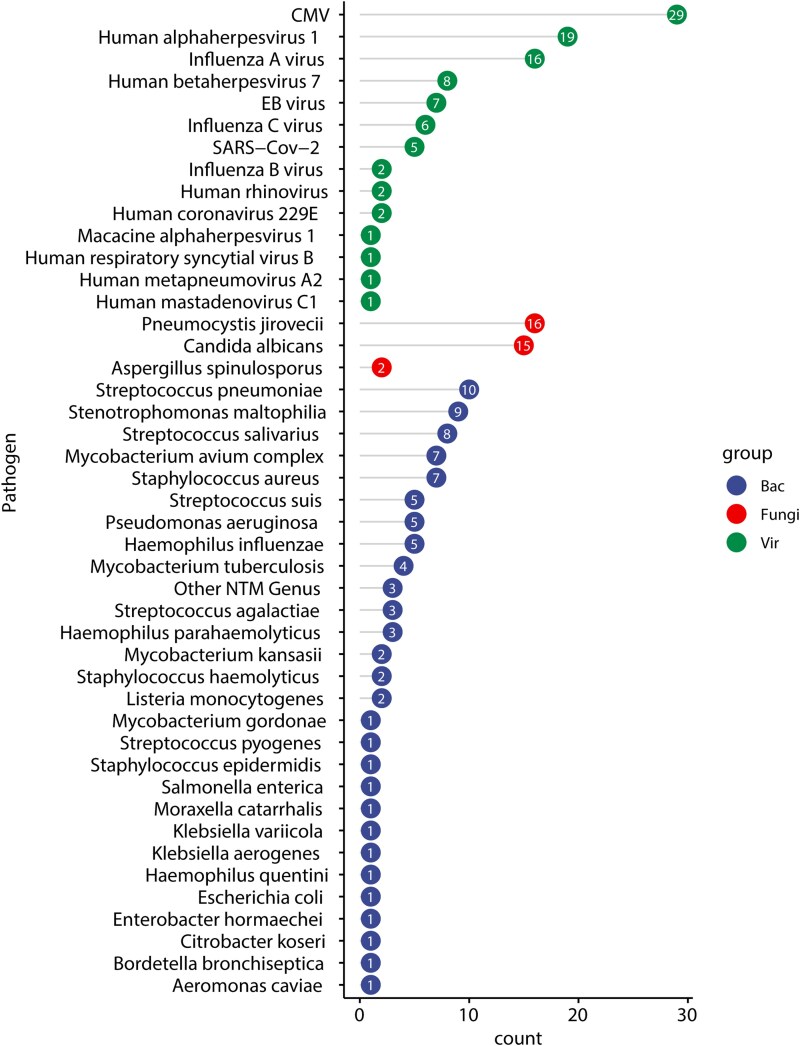
Pathogen species and detection frequency by mNGS.

Metagenomic next-generation sequencing (mNGS) identified 45 distinct pathogenic microorganisms, comprising 14 viruses, 3 fungi, and 28 bacterial species (Figure 3).

##### Bacterial Detection

*Streptococcus pneumoniae* exhibited the highest positivity rate (16.7%), followed by *Stenotrophomonas maltophilia* (15.0%), *Streptococcus salivarius* (13.3%), *Staphylococcus aureus* (11.7%), and *Mycobacterium avium* complex (11.7%). Notably, *M. tuberculosis* (MTB) demonstrated a lower detection rate by mNGS (6.7%) compared to CMT (11.7%), with a 75.0% concordance rate (3/4) against culture results (Table 2).

**Table 2.** Positivity Rates of CMT and mNGS for Major Pathogen Categories and Selected Pathogens

Category\Pathogen	*M. tuberculosis*	*P. jirovecii*	*C. albicans*	Cytomegalovirus	SARS-CoV-2
mNGS positivity rate	6.7%	26.7%	25.0%	48.3%	8.3%
CMT positivity rate	11.7%	26.7%	5.0%	15.0%	3.3%
Higher	CMT	Equal	mNGS	mNGS	mNGS

##### Fungal Detection

*Pneumocystis jirovecii* was detected at 26.7%, matching CMT's rate, while showing 56.3% concordance with clinical PCR and 100.0% with ddPCR. *Candida albicans* was identified in 25.0% of cases by mNGS—significantly higher than CMT (5.0%)—but with limited concordance (13.3% with culture; 40.0% with ddPCR) (Table 2).

##### Viral Detection

Cytomegalovirus (CMV) had the highest mNGS positivity rate (48.3%, vs 15.0% by clinical PCR), yet concordance was low (24.1%). Influenza A virus was detected in 26.7% of mNGS tests but showed 0% concordance with clinical PCR and only 6.3% with ddPCR. SARS-CoV-2 had an 8.3% mNGS detection rate (vs 3.3% by PCR), with 20.0% and 40.0% concordance to PCR and ddPCR, respectively (Table 2).

##### Overall Performance

mNGS demonstrated higher detection rates than CMT for bacteria (75.0%), fungi (45.0%), and viruses (80.0%). However, concordance varied widely, ranging from 75.0% for MTB (highest) to 0% for influenza A (lowest; note: only 1 PCR-positive case was available). Among 17 CMT-negative samples, mNGS detected pathogens in 13 (76.5%), including *C. albicans*, *S. maltophilia*, EBV, and respiratory syncytial virus (RSV). Clinical diagnoses in these cases aligned with findings (eg, bacterial-fungal pneumonia, PCP, and CMV pneumonia). However, the detection of C. albicans in BALF should be interpreted with caution, as colonization or contamination from the oral cavity or upper respiratory tract is common. In this study, we did not systematically document the presence of oral, esophageal, or tracheal candidiasis in our patients. Therefore, we are unable to distinguish between colonization and true infection. The high positivity rate and importance score for C. albicans may be overestimated.

#### Analysis of Consistency Between mNGS and CMT

The pathogenic microorganisms detected by BALF mNGS were clinically identified and compared with the CMT results to form the mNGS disease spectrum of AIDS combined with lung infection. The kappa consistency analysis showed that the mNGS disease spectrum was well consistent with the CMT disease spectrum, with a kappa value of 0.46 (between 0.40 and 0.75 indicates good consistency).

mNGS detected more than 2 pathogens in 85.7% of the samples, of which 33.3% detected a mixture of bacteria, fungi, and viruses, 45.8% detected a mixture of bacteria and viruses, and 10.4% detected a mixture of bacteria, fungi, and fungi and viruses. In addition, 3.6% of the samples reported only the detection of bacteria.

### Diagnostic Performance

The evaluation of diagnostic performance revealed significant differences between composite CMT and clinical diagnosis alone. For *M. tuberculosis* (MTB) detection using the mNGS RPMsample/no-template control (NTC) criteria, composite CMT demonstrated superior sensitivity (42.9%) compared to clinical diagnosis (21.4%), consistent with established literature. The composite approach also achieved better diagnostic accuracy (91.7% vs 80.0%). While traditional methods including culture and GeneXpert met clinical standards for MTB detection, mNGS showed limited sensitivity advantages but maintained excellent specificity (98.1%).

In *P. pneumonia* (PCP) detection, composite CMT outperformed clinical diagnosis across multiple parameters, showing higher sensitivity (58.3% vs 42.4%) and improved accuracy (80.0% vs 65.0%), with both methods demonstrating comparably high specificity. However, evaluation of other fungal infections (excluding PCP) revealed moderate sensitivity (58.0%) with composite CMT, potentially attributable to technical challenges in fungal cell wall disruption during sample processing.

Cytomegalovirus (CMV) detection presented a more complex pattern. Composite CMT showed marginally lower sensitivity (80.0% vs 81.8%) and accuracy (56.7% vs 63.3%) compared to clinical diagnosis, with both methods demonstrating suboptimal positive predictive value and specificity. Analysis of other viral infections revealed mNGS performance characteristics of 50.0% sensitivity and 65.5% specificity. These findings underscore the need for methodological improvements including optimization of viral reference databases through incorporation of specific reference sequences, refinement of experimental protocols, and implementation of virus-specific library preparation and sequencing workflows to enhance detection reliability and reduce false results.

The comprehensive performance assessment highlights both the capabilities and limitations of current diagnostic approaches, providing clear directions for technical optimization in pathogen detection methodologies.

### Clinical Correlation

**Figure 4.**
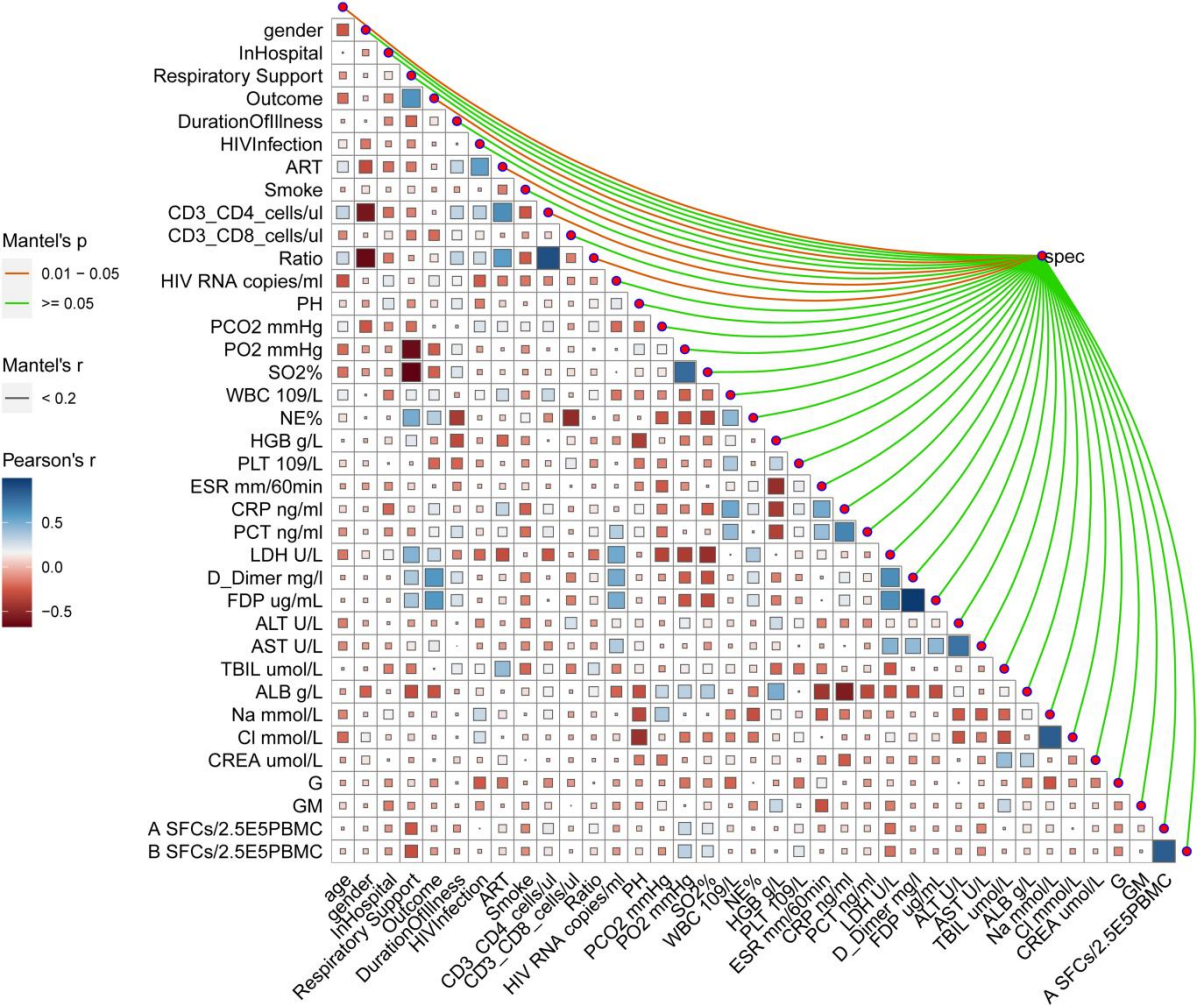
Correlation analysis between mNGS pathogen spectrum and clinical indicators.

Mantel test results showed that there was a significant correlation between the pathogen spectrum and age, outcome, ART, CD4+, and CD4/CD8 (*r* < 0.2, *P* < .05); however, no statistical correlation was observed with other clinical and immunological indicators. The correlation between clinical indicators was analyzed by Pearson chi-square test (Figure 4).

## DISCUSSION

The findings of this study suggest that mNGS provides a valuable adjunct in the etiological diagnosis of pulmonary infections in patients with AIDS. Compared with conventional microbiological testing (CMT), mNGS demonstrated broader pathogen detection capability for bacteria, fungi, and viruses, particularly in identifying mixed infections and complex pathogens, consistent with previous reports in the literature [10–12]. However, mNGS should be regarded as a complementary diagnostic tool rather than a substitute for established methods. This is particularly important for certain pathogens, such as viruses and *M. tuberculosis*, where conventional assays (eg, PCR, culture, and GeneXpert) remain essential, especially in resource-limited settings.

Although mNGS enables simultaneous detection of multiple microorganisms, the clinical relevance of identified pathogens requires careful interpretation in the context of clinical presentation and conventional test results [13–15]. The relatively low concordance observed in viral detection highlights several challenges, including incomplete reference databases, technical variability in sequencing workflows, and the potential for false-positive results due to contamination or colonization. In addition, the lower sensitivity of mNGS for *M. tuberculosis* further supports the continued reliance on conventional diagnostic approaches for tuberculosis. Therefore, mNGS should be integrated with, rather than replace, existing diagnostic strategies.

This study identified mixed infections in 55.2% of patients, with *P. pneumonia* (PCP) combined with bacterial infection being the most common pattern. Mixed infections pose substantial diagnostic and therapeutic challenges, particularly in severely immunocompromised patients. The broad-spectrum detection capability of mNGS may facilitate the identification of potential co-pathogens and provide additional diagnostic clues. However, these findings should primarily be interpreted as supporting clinical assessment and hypothesis generation rather than directly guiding treatment decisions. Further studies are needed to clarify the clinical significance of mixed infections and their impact on patient outcomes.

Despite limitations related to the COVID-19 pandemic, including a relatively small sample size and the inability to assess real-time treatment adjustments based on mNGS results, this study provides a comprehensive characterization of the pathogen spectrum in pulmonary infections among patients with AIDS. By systematically comparing mNGS, conventional microbiological testing, and clinical diagnoses, this study contributes to a better understanding of the potential role of mNGS in clinical practice.

Importantly, the clinical utility of mNGS in this context should be interpreted as supporting diagnostic evaluation, risk stratification, and hypothesis generation, rather than serving as a standalone basis for therapeutic decision-making. These findings may inform future studies aimed at optimizing the integration of mNGS into routine clinical workflows.

The lower concordance of mNGS in viral detection underscores the need for further refinement of viral reference databases and standardization of experimental workflows, including improvements in sequence selection and contamination control. In addition, the machine learning approach used in this study, specifically the random forest analysis, should be interpreted as exploratory in nature. Its primary purpose was to prioritize potentially clinically relevant pathogens rather than to establish causal relationships.

Future studies with larger sample sizes and prospective designs are warranted to validate these findings and to further evaluate the clinical applicability and robustness of mNGS in real-world settings.

## CONCLUSIONS

Overall, our findings suggest that mNGS may serve as a complementary tool in the etiological evaluation of pulmonary infections in patients with AIDS, rather than a standalone replacement for conventional testing. Its limitations in viral interpretation and *M. tuberculosis* diagnosis still need to be addressed. In addition, because the present study did not systematically evaluate treatment modification, treatment outcomes, length of hospital stay, or mortality in relation to mNGS findings, the clinical impact of mNGS should be interpreted cautiously. Future research should focus on optimizing technical workflows, expanding sample size, and integrating more detailed clinical outcome data to better define the role of mNGS in clinical practice.
